# Knowledge, attitudes and beliefs about the health hazards of biomass smoke exposure amongst commercial food vendors in Nigeria

**DOI:** 10.1371/journal.pone.0191458

**Published:** 2018-01-29

**Authors:** Ogonna N. O. Nwankwo, Ndubuisi Mokogwu, Orighomisan Agboghoroma, Fahmi O. Ahmed, Kevin Mortimer

**Affiliations:** 1 Department of Community Medicine, University of Calabar Teaching Hospital, Calabar, Nigeria; 2 Department of Community Health, University of Benin Teaching Hospital, Benin, Nigeria; 3 Department of Medicine, Jos University Teaching Hospital, Jos, Nigeria; 4 College Health Sciences, Addis Ababa University, Addis Ababa, Ethiopia; 5 Department of Clinical Sciences, Liverpool School of Tropical Medicine, Liverpool, United Kingdom; Universita degli Studi della Tuscia, ITALY

## Abstract

**Background:**

Exposure to biomass smoke is a major cause of morbidity and mortality in Africa. Commercial food vendors in Nigeria and elsewhere in Africa are commonly exposed to biomass smoke from open fire cooking both at work and home. Little is known about the knowledge, attitudes and beliefs of food vendors about the health hazards of biomass smoke exposure in Nigeria.

**Methods:**

We did a descriptive cross sectional survey of the knowledge, attitudes and beliefs of commercial food vendors in the cities of Benin and Calabar in Nigeria. We recruited respondents using a multi-stage approach. Structured interviewer-administered questionnaires were used for data collection.

**Results:**

We recruited 308 participants (164, 53.2% female). The majority 185(60.2%) were married and had post-primary education 206(67.4%). The average monthly income was <30,000 Naira (US$150). Most 198(64.4%) were not aware that biomass smoke exposure is harmful to human health. About three-quarters (221; 71.8%) were unconcerned as to the effect of exposure to fumes from biomass fuels on their health. Less than half of respondents (110, 41.6%) believed biomass smoke was harmful to health. Male gender, being single, having post-primary education and preferring electricity or gas fuels were associated with good knowledge of the adverse health effects of biomass smoke exposure whilst female gender and having good knowledge of the adverse health effects of biomass smoke were associated with positive attitudes towards preventing exposure.

**Conclusion:**

Commercial food vendors in our study had limited knowledge about the adverse health effects of biomass smoke exposure and negative attitudes towards preventing these adverse health effects. We suggest an educational intervention is needed to improve this knowledge.

## Introduction

Approaching half of the World’s population depend on biomass fuels (animal dung, crop residues, wood, charcoal) for their day-to-day fuel energy needs.[1] Biomass fuel use is a major cause of both indoor (household) and ambient (outdoor) air pollution with a socio-economic differential in its use. [2–4] It is now widely recognized that exposure to smoke from the burning of biomass fuels is a major cause of global morbidity and mortality from respiratory diseases and non-communicable diseases such as lung cancer, stroke, ischaemic heart diseases amongst others.[3–9] Pneumonia in children and chronic obstructive pulmonary disease (COPD) in adults have been most clearly associated with biomass smoke exposure.[7, 10–12] Dependence on biomass fuels is a problem of poverty with low-middle income country (LMIC) populations, especially the poorest of the poor, bearing a disproportionate burden of the attributable diseases. [8, 13] More than one third of the total burden of disease attributed to biomass smoke is seen in Africa. [14, 15]

Commercial food vending is a popular business activity in most cities in Africa. [16–18] Commercial food vendors typically cook street foods like bean cake, roasted plantain, bean pudding and local delicacies using biomass fuels burned in open fires. Commercial food vendors are often women who are often also the primary cooks at home and as such experience frequent biomass smoke exposure through these gender-based roles.[18–20] There is evidence from studies done in Nigeria and Cameroon that there is a higher prevalence of respiratory symptoms in commercial food vendors and cooks exposed to biomass smoke compared to those who are not exposed.[19, 21–23] Again, due to the nature of their work, they are exposed to other sources of air pollution such as vehicular emission, stationary power generating sets amongst others which further predisposes them to health hazards. [2, 4]

Behavioural and social medicine has postulated a number of theories in trying to explain and influence human actions, of which one of the most important is the social cognitive theory which offers possible explanation on the relationship between one’s attitude and behaviour, with knowledge given a special importance due to its mediating role.[24–27] This theory amongst others forms the underpinning for the traditional knowledge–attitude–behavior models (KAB) or sometimes KAP model (referring to knowledge, attitude and practice) on changing actions.[28, 29] Various studies from health related issues have shown the important mediating role knowledge plays for environmental and health-related attitude, behavior and practise.[25, 30–35]

Improving knowledge about the harmful health effects of biomass smoke has been proposed as an important step towards modifying behavior to reduce exposure to biomass smoke and other forms of household air pollution.[36] A lack of knowledge about the link between exposure to biomass smoke and adverse health outcomes as well as acceptability and accessibility of clean energy has been found to be important barriers to the uptake of clean cooking technologies.[37, 38]

Little is known about levels of knowledge, attitudes and beliefs of food vendors about the adverse health effects of biomass smoke exposure in African countries like Nigeria. We set out to fill this gap in the evidence by undertaking a cross-sectional survey of commercial food vendors in Benin and Calabar cities, Nigeria.

## Materials and methods

### Setting

Nigeria is located on the western coast of Africa and occupies approximately 923,768 square kilometres of land stretching from the Atlantic coast in the south to the fringes of the Sahara Desert in the north.[39] It is bounded by the republics of Niger and Chad in the north, the Republic of Cameroon on the east, and the Republic of Benin on the west. The country’s last population and housing census in 2006 is placed at over 140 million which is projected to be 187,648,291 in 2017.[39, 40] The country has a tropical climate with wet and dry seasons and has varying vegetation ranging from mangrove swamp forest in the south to Sahel grassland in the north. It has over 100 ethnic groups with Igbo, Yoruba and Hausa-Fulani groups being the commonest ones. Nigeria runs a democratic system of government and is made up of 36 states with the federal capital territory located in Abuja. Benin and Calabar cities are in 2 different states in the South-south geo-political zone of Nigeria. Both cities represent typically most big cities in Nigeria and their residents are mainly small- to medium-scale business owners, farmers, artisans, civil servants, bankers, and students. The country’s gross national income per capita which reflects the average income of citizens of the country for the year as of 2016 is US$2450 while the poverty threshold level per individual is set at US$276 per annum. [40, 41]

### Participants

A multi- stage sampling method was used to select the respondents. A single local government area (LGA) was initially selected from the LGAs that made up each city (3 LGAs made up Benin city while 2 LGAs made up Calabar city) by simple random sampling using balloting. Thereafter two wards were selected from a list of the wards that make up the selected LGAs (12 wards for Oredo LGA in Benin city and 10 wards for Calabar Municipality LGA in Calabar city) using simple random sampling technique. All commercial food vendors found in the streets making up the selected wards were eligible to participate. Out of 337 potential respondents found in the wards we sampled, 308 agreed to participate with most declining on account of the busyness of their schedule. Most of the respondents as stated in previous study operate mainly small to medium outlets with some cooking in enclosures and vending their meals ranging from fried or smoked fish and meat, roasted corn and pears, fried yam and potatoes, bean cake as well as local delicacies outside.[16, 20, 42]

### Data collection

Data was collected between September and October 2015, using a structured interviewer-administered paper questionnaire partly based on the standardized American thoracic society-division of Lung disease (ATS-DLD) questionnaire and literature reviews.[43, 44]The survey instrument was reviewed by a group of academics, occupational health physicians, epidemiologists and public health physicians drawn from academic and health institutions from both cities as well as environmental health experts from Liverpool school of Tropical Medicine and found to have face validity. The reviews resulted in minor modification in the initially designed questionnaire. The questionnaire had 3 sections: 1) socio-demographic data including age, occupation, household size, income and marital status; 2) the respondents’ knowledge about biomass smoke and associated health problems; 3) questions about attitudes and beliefs towards the adverse health effects of biomass smoke. The details of the specific questions that were asked are in S1 File. A composite knowledge and attitude score was derived by applying a scoring system to questions pertaining to knowledge (Q15, Q17, Q18, Q20 and Q25) and attitudes (Q26, Q28, Q30, Q33 and Q34) in the questionnaire. One point was awarded for each correct answer and no point was awarded for an incorrect answer both for the single choice and multi-choice question types. Questions with Likert scale questions were ranked such that stronger agreement with positive answers had higher score with a one-point interval between each rank.

The questionnaire was pre-tested among 20 traders from neighbouring LGAs to our study sites to ensure comprehensibility, clarity of wording, and reliability of study tool. Three trained research assistants from each site, who had a minimum of university degree were trained to do the data collection.

### Sample size

Sample size was calculated using a single proportion sample size formula assuming 14% of participants would have a given level of knowledge based on the findings of a previous study,[44] a precision of 5%, clustering factor of 1.5 and a 10% non-response rate. This gave a target sample of 300.

### Statistical analysis

Questionnaire data were entered into the IBM statistical package for scientific solution (SPSS) version 20 statistical software (SPSS Inc, Chicago, IL, USA) in preparation for analysis. The primary outcome was a composite measure of the levels of the knowledge and attitudes about the adverse health effects of biomass smoke exposure. The composite scores calculated for each respondent was converted to percentages such that scores of less than 50% was categorized as poor knowledge or attitude while a score of 50% and above was categorized as good knowledge or attitude respectively. Categorical data were presented as frequency tables while inferential analysis was done at bivariate level with Chi-square test and at multivariate level with binary logistic regression. The level of statistical significance was set at P < 0.05.

### Ethical considerations

The research ethics committees of the University of Benin Teaching Hospital and the University of Calabar Teaching Hospital, Nigeria approved the study. The protocol numbers were ADM/E 22/A/VOL. VII/1239 and UCTH/HREC/33/366. Written informed consent was obtained from all respondents. For respondents considered minors consent was obtained from their parents or guardians while assent was obtained from them.

## Results

We interviewed 308 commercial food vendors, 106 (34.4%) from Benin City and 202 (65.6%) from Calabar. The mean (SD) age was 35.9 (10.8) years; 164 (53.2%) were women (Table 1). Half of the vendors (167; 54.2%) were educated to secondary level. A quarter (75; 24.4%) had worked at their present job for six to ten years. More than two thirds of the vendors (215; 69.8%) earned less than twenty thousand naira (100 US$) a month (US$1 = **₦**200). The median (range) number of days spent working each week was 6 (0–7) and the median (range) amount of time spent cooking at work and at home per day was 3 (0–10) and 2 (0–8) hours, respectively.

**Table 1:** Food vendor characteristics.

Variable	n(%) n = 308
**Age Group**	
16–25	54(17.5)
26–35	108(35.1)
36–45	93(30.2)
46–55	36(11.7)
>55	17(5.5)
**Sex**	
Male	144(46.8)
Female	164(53.2)
**Marital status**	
Single	91(29.5)
Married	185(60.1)
Widowed	13(4.2)
Separated/divorced	13(4.2)
Co-habiting	6(1.9)
**Level of education**	
No formal	26(8.4)
Primary	76(24.7)
Secondary	167(54.2)
Tertiary	33(10.7)
Postgraduate	6(1.9)
**Monthly income (₦)**	
<10000	112(36.4)
10000–19999	103(33.4)
20000–29999	56(18.2)
30000–39999	19(6.2)
40000–49999	13(4.2)
50000 and above	5(1.6)
**Duration in present job (years)**	
0–5	208(67.5)
6–10	75(24.4)
11–15	13(4.2)
>15	12(3.9)
Fuel type used*	
Biomass fuel	182(63.0)
Kerosene	131(45.3)
Gas	26(9.0)
Electricity	2(0.7)

* Multiple responses

### Knowledge, attitudes and beliefs amongst the food vendors about the harmful effect of exposure to biomass fuel smoke

A third of vendors (110; 35.7%) reported being aware of the harmful effects of biomass smoke exposure; respiratory problems such as cough and catarrh were most commonly reported. An awareness of breathing problems in children exposed to biomass fuel smoke was reported by 107 (34.7%) participants. Methods reported by the respondents to mitigate the harmful effect of exposure to biomass fuel smoke included using a room with open doors and windows as a kitchen (147; 61.0%), use of a separate room for cooking (142; 58.9%), shielding the fire (127; 52.7%) and using a cleaner cooking fuel (63; 26.1%). A composite score of knowledge revealed that 169 (29.9%) vendors had good knowledge of the harmful effects of biomass smoke exposure.

Regarding attitudes, 87 (28.2%) vendors reported they would be concerned if informed that their current cooking fuel could adversely affect their health; the remainder (221; 71.8%) did not know, were indifferent or were unconcerned. Just under half (146; 47.4%) perceived their cooking fuel as safe for their health; 11 (3.65) and 102 (33.1%) perceived their cooking fuel as highly unsafe and unsafe to their health, respectively. One hundred and forty (45.5%) vendors agreed to potentially support a ban on the commercial use of cooking fuels that emit harmful levels of smoke if government was to embark on that. A composite score of attitudes revealed that 90 (29.2%) vendors had positive attitudes towards preventing the adverse health effects of biomass smoke exposure.

Regarding beliefs, 192 (62.3%) vendors believed that some forms of cooking fuel such as kerosene, electricity were less harmful to health than others. Liquid petroleum gas (LPG) was believed to be the least harmful by 123 (64.1%) of these vendors while biomass fuel was believed to be least harmful by only 7 (3.6%). Of the 180 vendors who gave a reason for their belief that LPG was least harmful, 84 (46.7%) said this was because LPG is smokeless. When asked about their preferred cooking fuel, kerosene was reported by 107 (34.7%), gas by 87 (28.2%) and biomass by 86 (27.9%) vendors. The reasons vendors chose biomass fuels were due to low cost (n = 66, 76.7%), fast cooking time (n = 65, 75.6%) and the better taste (n = 58, 67.4%).

Bivariate analysis of knowledge score against independent variables revealed that knowledge was significantly associated with male gender (p<0.001), a higher level of education (p<0.001), monthly income (p = 0.001), preferred cooking fuel source (p = 0.03) and duration spent in present job (p = 0.001) (Table 2).

**Table 2:** Bivariate variables for knowledge of harmful effects of biomass fuel smoke.

Predictor variables	Knowledge n(%)		p value	OR (95% CI)
	Poor	Good		
**Age group**				
16–25	40(18.5)	14(15.2)	0.275	1
26–35	75(34.7)	33(35.8)		1.26(0.61–2.60)
36–45	68(31.5)	25(27.2)		1.05(0.49–2.24)
46–55	25(11.6)	11(12.0)		1.26(0.50–3.16)
>55	8(3.7)	9(9.8)		3.21(1.07–9.69
**Sex**				
Female	135(62.5)	29(31.5)	0.0001*	1
Male	81(37.5)	63(68.5)		0.28(0.16–0.46)
**Marital status**				
Ever married	155(71.8)	56(60.9)	0.062	1
Never married	61(28.2)	36(39.1)		0.61(0.37–1.02)
**Level of education**				
No formal	24(11.1)	2(2.2)	0.0001*	1
Primary	63(29.2)	13(14.1)		2.48(0.53–11.48)
Secondary	118(54.6)	49(53.3)		4.98(1.17–21.31)
Tertiary/PG	11(5.1)	28(30.4)		30.55(0.40–4.29)
**Monthly income (₦)**				
<10000	92(42.6)	20(21.7)	0.001*	1
10000–19999	69(31.9)	34(37.0)		2.27(1.21–4.26)
20000–29999	33(15.3)	23(25.0)		3.21(1.57–6.55)
30000–39999	8(3.7)	11(12.0)		6.32(2.31–17.34)
≥40000	14(6.5)	4(4.3)		1.31(0.40–4.29)
**Preferred cook fuel**				
Biomass	71(32.9)	15(16.3)	0.001*	1
Kerosene	81(37.5)	26(28.3)		1.52(0.75–3.08)
Gas	49(22.7)	38(41.3)		3.67(1.83–7.36)
Electricity	15(6.9)	13(14.1)		4.10(1.64–10.25)
**Duration in present job**				
0–5	150(69.4)	58(63.0)	0.032*	1
6–10	54(25.0)	21(22.8)		1.01(0.56–1.81)
11–15	8(3.7)	5(5.4)		1.62(0.53–4.93)
>15	4(1.9)	8(8.7)		5.17(1.58–16.97)

*Statistically significant at p<0.05 and the absence of the null value (1.00) from the CIs of the odds ratio.

Bivariate analysis of attitude composite score against independent variables revealed that positive attitudes to preventing adverse health effects of biomass smoke exposure were associated with older age (p = 0.048), female sex (p<0.01), being married (p = 0.012), higher monthly income (p<0.01) and having knowledge about the adverse health effects of biomass smoke exposure (p<0.01) (Table 3).

**Table 3:** Bivariate variables for good attitude towards preventing harmful effects of biomass fuel smoke.

Predictor variables	Attitude		P-value	OR (95% CI)
	Positive	Negative		
**Age group**				
16–25	10(18.5)	44(81.5)	0.048*	1
26–35	35 (32.4)	73(67.6)		2.11(0.96–4.65)
36–45	31(33.3)	62(66.7)		2.20(0.98–4.92)
46–55	13(36.1)	23(63.9)		2.49(0.96–6.46)
>55	1(5.9)	16(94.1)		0.27(0.03–2.19)
**Sex**				
Female	69(42.1)	95(57.9)	0.0001*	1
Male	21(14.6)	123(85.4)		4.25(2.44–7.41)
**Marital status**				
Ever married	71(33.6)	140(66.4)	0.012*	1
Never married	19(19.6)	78(80.4)		2.08(1.17–3.70)
**Level of education**				
No formal	8(30.8)	18(69.2)	0.133	1
Primary	29(38.2)	47(61.8)		1.39(0.54–3.54)
Secondary	46(27.5)	121(72.5)		0.86(0.35–2.07)
Tertiary/PG	7(17.9)	32(82.1)		0.49(0.16–1.55)
**Monthly income (₦)**				
<10000	34(30.4)	78(69.6)	0.005*	1
10000–19999	26 (25.2)	77(74.8)		0.77(0.43–1.28)
20000–29999	15(20.8)	41(73.2)		0.84(0.41–1.71)
30000–39999	3(26.8)	16(84.2)		0.43(0.12–1.52)
≥40000	12(66.7)	6(33.3)		4.59(1.63–12.89)
**Duration in present job**				
0–5	58(27.9)	150(72.1)	0.222	1
6–10	27(36.0)	48(64.0)		1.45(0.83–2.54)
11–15	4(30.8)	9(69.2)		1.15(0.36–3.71)
>15	1(8.3)	11(91.7)		0.24(0.03–1.71)
**Knowledge of harmful effects**				
Poor	80(88.9)	136(62.4)	0.0001*	1
Good	10(11.1)	82(37.6)		0.21(0.10–0.42)
**Preferred cook fuel**				
Biomass	28(32.6)	58(67.4)	0.483	1
Kerosene	33(30.8)	74(69.2)		1.08(0.59–1.99)
Gas	24(27.6)	63(72.4)		1.27(0.66–2.42)
Electricity	5(17.9)	23(82.1)		2.22(0.78–6.35)

*Statistically significant at p < 0.05 and the absence of the null value (1.00) from the CIs of the odds ratio.

A logistic regression analysis to control for confounders and determine predictors of knowledge about the adverse health effects of biomass smoke exposure and attitudes towards preventing adverse health effects of biomass smoke exposure is shown in Table 4. The analysis revealed that being male, single, having post-primary education, having a preference for electricity or gas as a source of fuel and earning about 10,000 Naira (US$50) were significantly associated with higher levels of knowledge about the adverse health effects of biomass smoke exposure. Being female and having a high knowledge score were significantly associated with having a positive attitude towards preventing adverse health effects of biomass smoke exposure.

**Table 4:** Logistic regression for predictors of knowledge and attitude of food vendors.

Variable	OR (95% CI)	p value
**KNOWLEDGE**		
**Age group**		
16–25	1	
26–35	1.662(0.570–4.620)	0.365
36–45	1.805(0.486–6.702)	0.377
46–55	2.979(0.595–14.916)	0.184
>55	5.032(0.681–37.184)	0.113
**Sex**		
Male	1	0.027*
Female	0.490(0.260–0.924)	
**Marital status**		
Never married	1	
Ever married	0.232(0.097–0.555)	0.001*
**Educational level**		
No formal	1	
Primary	2.082(0.381–11.392)	0.398
Secondary	5.319(1.017–27.811)	0.048*
Tertiary/PG	23.371(3.505–155.841)	0.001*
**Preferred fuel**		
Biomass	1	
Kerosene	1.062(0.433–2.604)	0.896
Gas	2.676(1.086–6.593)	0.032*
Electricity	3.568(1.089–11.690)	0.036*
**Monthly income**		
**<** 10000	1	
10000–19999	2.798(1.233–6.352)	0.014*
20000–29999	2.113(0.764–5.843)	0.149
30000–39999	2.271(0.526–9.809)	0.272
40000≥	0.906(0.177–4.622)	0.905
**Duration in present job**	1.089(0.987–1.201)	0.088
**ATTITUDES**		
**Age group**		
16–25	1	
26–35	1.544(0.583–4.088)	0.382
36–45	1.183(0.367–3.810)	0.779
46–55	0.928(0.215–4.004)	0.920
>55	0.133(0.10–1.784)	0.128
**Sex**		
Male	1	
Female	3.179(1.646–6.140)	0.001*
**Knowledge of harmful effects**		
Good	1	
Poor	0.278(0.117–0.658)	0.004*
**Marital status**		
Never married	1	
Ever married	1.567(0.720–3.412)	0.258
**Educational level**		
No formal	1	
Primary	1.402(0.480–4.092)	0.537
Secondary	0.850(0.296–2.441)	0.763
Tertiary/PG	1.050(0.235–4.678)	0.949
**Preferred fuel**		
Biomass	1	
Kerosene	1.425(0.680–2.989)	0.348
Gas	1.719(0.736–4.012)	0.210
Electricity	0.773(0.230–2.603)	0.678
**Monthly income**		
**<** 10000	1	
1000–19999	0.975(0.475–1.999)	0.944
20000–29999	1.334(0.538–3.307)	0.534
30000–39999	0.840(0.173–4.085)	0.829
40000≥	6.752(1.722–26.483)	0.006
**Duration in present job**	1.018(0.926–1.120)	0.708

*Statistically significant and the absence of the null value (1.00) from the CIs of the odds ratio.

## Discussion

The main findings of our cross-sectional study are that over half the Nigerian commercial food vendors we sampled were unaware of the adverse health effects of biomass smoke exposure, and an even greater proportion, about three-quarters, had poor attitudes towards preventing these adverse health effects and were unconcerned as to whether it is harmful to their health or not. This was despite the majority study participants believing that biomass smoke exposure is harmful.

We found that determinants of higher levels of knowledge about the adverse health effects of biomass smoke exposure were being male, single, having a post- primary level of education and having a preference for electricity or gas as a source of fuel whilst being female and having good knowledge about the harmful effects of biomass smoke were independently associated with positive attitudes towards preventing exposure.

Our findings are consistent with four previous studies that found low levels of knowledge and poor attitudes about the adverse health effects of biomass smoke or other sources of household air pollution amongst people using biomass fuels for their household energy needs. [11, 45–47].Low levels of knowledge may imply that more health education needs to be done by public health personnel to educate individuals on the harmful effects of biomass smoke.

However, our results contrast with two other studies from different settings which found that there was a high knowledge about the harmful health effects of biomass smoke. The explanation for this inconsistency in the literature may be the smaller sample size used and the purposive nature of sampling in the latter studies. [46–49]

Edelstein and colleagues found a higher proportion of people with positive attitudes about the adverse health effects of biomass smoke compared to our current study although this was a pilot study with limited sample size and included participants from both rural and urban areas.[44] Our findings are consistent with an earlier study done in a rural community which revealed poor attitudes towards reducing household air pollution from sources such as biomass smoke exposure amongst the respondents.[47] Furthermore, studies in other low-middle income countries have shown that having higher levels of education predict better knowledge about the adverse health effects of biomass smoke similar to our findings.[50, 51] It is believed that there is a strong link although not direct between education and disease. This implies the need for advocacy on the need for improved general education in the population. Knowledge of the harmful effects of biomass fuel was a significant predictor of positive attitudes in our study. This is in keeping with a study carrying out in Malaysia looking at attitudes to a form of air pollution which showed some level of correlation between knowledge and attitudes.[29]This is another strong pointer to the need for education, especially to groups of individuals who are exposed to these hazards in the course of their daily trade. Special and routine trainings and education needs of this occupation groups should be a priority to public health officials. In addition, preventing exposure to biomass fuel is a very important principle of prevention. An example of this principle is replacing the use of biomass fuels with less harmful fuel such as cooking gas or electricity. This types of fuels are however more expensive to purchase. This study revealed that persons who used cooking gas and electricity had significantly better knowledge of the harmful effects of biomass fuel. In addition, earning a larger income significantly improved attitudes towards the harmful effects of biomass fuel. This is a strong indication that having more economic power improves health seeking behaviours. Therefore, local authorities need to prioritize on providing cost effective and cleaner cooking fuels to these food vendors who are mostly in the lower socio-economic class.

Strengths of our study include the large sample size from two cities, the inclusion of men and women, and a wide age range. The study would have been strengthened by including qualitative research methods such as in-depth interviews and focus group discussions. This would have given the opportunity to explore from the respondent’s perspectives on other socio-cultural issues that can influence the level of knowledge and attitudes to biomass smoke exposure.

A limitation of this study was that responses made by the food vendors were based on self-report and it was difficult to authenticate some of their responses. The inclusion of objective assessments of vendors’ exposure to biomass smoke and their respiratory health would have added additional value. Other limitation of our current work was the non-measurement of their knowledge and attitudes to other sources of outdoor air pollution as well as the level of outdoor air pollution the food vendors may have been exposed to. This could potentially influence their knowledge and attitudes to biomass smoke and other sources of air pollution.

It is recommended that future studies should explore the knowledge and attitudes of food vendors to other environmental sources of air pollution that their occupation predispose them to and its potential effect on their health using a mixed method approach.

Our study has demonstrated that, despite the evidence available regarding the health effects of biomass fuel smoke, the message may still be largely within the domain of researcher and policy makers. This is of great concern as awareness and knowledge of this issue is necessary to change behaviours towards adoption of cleaner sources of energy and reducing household air pollution exposure.

In conclusion, we found that Nigerian commercial food vendors have limited knowledge about the health hazards of their occupation-associated use of biomass fuels and have negative attitudes towards preventing these adverse effects despite high levels of belief that these fuels are harmful to health. There is a need for evidence-based interventions such as implementing health education campaigns using compelling messages distributed via short-message-services (SMS),vendor’s involvement in air quality monitoring reading and interpretation, conditional cash transfer to improve knowledge, attitudes and beliefs about the adverse health effects of biomass smoke and to develop effective and cost-effective alternatives to biomass fuels for commercial food vendors in Nigeria and elsewhere in Africa.

## Supporting information

S1 File File containing study questionnaire. (DOC)

S2 File File containing dataset. (XLS)
