# Supplementary material for: Knowledge, attitudes and beliefs about the health hazards of biomass smoke exposure amongst commercial food vendors in Nigeria
Source: PLoS One. 2018 Jan 29;13(1):e0191458. doi: 10.1371/journal.pone.0191458 (PMC5788333; doi:10.1371/journal.pone.0191458)
Supplement: S1 File — (DOC) [file pone.0191458.s001.doc]

**S1 File. File containing Study Questionnaire**

**INFORMED CONSENT (**Introduction to respondents and consent)

My name is………I am a research assistant working for Dr Nwankwo and Dr. Mokogwu , staffs of the University of Calabar Teaching Hospital, Calabar and University of Benin Teaching Hospital,Benin who are conducting a research to assess the knowledge, attitudes and beliefs of food vendors to biomass fuel smoke in Calabar and Benin cities of Nigeria. The project seeks to assess the awareness of people who vend food in different forms on the street about the health effect of the use of biomass fuel (such as wood, crop residues, charcoals, e.t.c) as well as their attitudes and beliefs to the smoke that comes from it. The findings of the work would be used in helping to advocate for the improvement of health of people involved in such occupations as well as in development of policies geared towards better environmental health for all.

It is important for you to understand that your participation in this study is completely voluntary. We would be really grateful if you would agree to participate in this study, but do feel free to refuse. If you refuse, there will be no consequence for you.If you choose to participate in this study you need to know that you may withdraw from the study at any stage without giving any explanation for your withdrawal.

The information you provide will be confidential. We will not use your name on any publications or write up from this study. There is no immediate benefit from the study however its finding can help in informing policies to improve health for everyone.There are no known risks to you resulting from your participation in the study. The interview will take maximum of 30 minutes. If you experience any personal discomfort, you may, as stated above, stop at any time or refuse to answer any questions.

For more information,Contact Dr Nwankwo directly on:phone number(deleted), email(deleted).

In case of any inconveniences / ethical concerns about the study, you can contact:

The office of the Chairman, Health Ethics and Research Committee,

University of Calabar Teaching Hospital, Calabar, Cross River State/University of Benin Teaching Hospital,Benin city Edo State.

**Part II: Certificate of Consent**

Do you have any questions? Do you want to participate? (Circle) Yes / No

*If Yes:* Please give your consent as indicated below.

I have read the foregoing information, or it has been read to me. I have had the opportunity to ask questions about it and any questions that I have asked have been answered to my satisfaction. I consent voluntarily to participate as a participant in this research.

Signature of Participant__________________ Date _____________________

*****If respondents a minor(less than 18 years, first get the consent from the parent/guardian and then get assent from the minor)*

Researcher / Research Assistant

I confirm that the participant was given an opportunity to ask questions about the study, and all the questions asked by the participant have been answered correctly and to the best of my ability. I confirm that the individual has not been coerced into giving consent, and the consent has been given freely and voluntarily.

 Name and signature of Researcher/person taking the consent_____________________

Date__________________________ (Day/month/year

**Knowledge, Attitude and Belief of Street food vendors in Nigeria towards Biomass smoke fuel questionnaire**

**Section A: Socio-demographic data**

1. Age as at last birthday (years)........................
2. Sex Male ( ) Female ( )
3. Marital status: Single ( ) Married ( ) Widowed ( ) Separated/Divorced( ) Co-habiting()
4. Level of Education: No formal( ) Primary education( ) Secondary education()

Tertiary education ( ) Postgraduate education ( )

1. Religion: Christianity( ) Islamic( ) African Traditional Religion ( )

Others (specify)......................................................

1. Average income per month: <10,000 [ ] 10,000-19,999[ ] 20,000-29,999 [ ]

30,000-39,999 [ ] 40,000-49,999[ ] 50,000 and above[ ]

1. How long have you been employed in your current place of work? .......................................
2. What is your job description? ......................................................................................................
3. Length of time spent in cooking at home in a day (hours)? .........................................................
4. Length of time spent in cooking at work place in a day (in hours)? ......................................
5. Number of days a week spent at work?.................................................
6. What type of cooking fuels do you use at home? (Tick as many)

Biomass fuel (firewood, charcoal, animal dungs[ ] Kerosene[ ] Gas[ ] Electricity[ ] Others(specify).............................................................................

1. What type of cooking fuels do you use at work? (Tick as many)

Biomass fuel (firewood, charcoal, animal dungs[ ] Kerosene[ ] Gas[ ] Electricity[ ] Others(specify).............................................................................

1. .Have you ever worked in any of the following places:

Coal mine? Yes ( ) No ( )

Quarry? Yes ( ) No ( )

Pottery? Yes ( ) No ( )

Block moulding industry Yes ( ) No ( )

Asbestos making plant Yes ( ) No ( )

Glass, ceramic or abrasive manufacturing plant? Yes ( ) No ( )

Others (specify) ................................................

**Section B: Knowledge of cooking smoke and health problems.**

1. Do you know that Biomass smoke(cooking smoke from plant products such as firewood, leaves, charcoal, etc. or animal products like animal dungs) can causes serious medical conditions? Yes ( ) No ( ) Don’t know ( )
2. If yes, how? ................................................................................................................................

....................................................................................................................................................

1. The following are adverse effects of exposure to biomass cooking smoke? (Tick as many)

Respiratory problems like cough, catarrh [ ]

Wheeze and difficulty breathing or breathlessness [ ]

Pregnant women delivering babies with low birth weight [ ]

Cancer [ ]

Cataracts [ ]

Pulmonary tuberculosis [ ] Others(specify)......................................................................

1. Can biomass cooking smoke cause Breathing (or chest) problems in children exposed to the smoke? Yes ( ) No ( ) Don’t know ( )
2. If yes, explain:......................................................................................................
3. Can cooking smoke causes breathing problems in people who do the cooking?

Yes ( ) No ( ) Don’t know ( )

1. If yes, explain............................................................................................................
2. Do you think your current source of cooking fuel smoke can have a potential negative health effect on you? Yes ( ) No ( ) Don’t know ( )
3. If yes, what are these effects? i)......................................................................

ii)......................................................................

iii)....................................................................

1. What is the source of your information on the health effects of cooking smoke?

Television ( ) Radio ( ) Internet ( ) Newspapers ( ) Family ( ) Friends ( )

Neighbours ( ) workshops ( ) Health workers ( ) Religious places ( )

1. How can you protect yourself from the negative health effects of cooking smoke?(Tick several)

Using fan ( )

Shielding fire ( )

Wearing a face cover, handkerchief ( )

Cooking in a room with open windows or doors ( )

Use of cleaner fuels (gas or electricity) ( )

Having a separate room for cooking ( )

Others (specify) .....................................................

Don’t know ( )

**Section C: Attitude**

1. Would you be concerned if you are you are told your current cooking fuel smoke affects health? Not concerned( ) Indifferent ( ) Concerned( ) Don’t know( )
2. Reasons for answer above?.......................................................................................
3. If you are told that your cooking fuel smoke (biomass cooking smoke) is bad for your health, would you be willing to do something about it?

Strongly unwillingly[ ] Unwillingly[ ] Indifferent[ ] Willingly[ ] Strongly willingly[ ]

1. If willing what would you do? ............................................................................................................

................................................................................................................................

1. If you are told that biomass cooking smoke can cause Breathing (or chest) problems in people who cook with it or children exposed to it, would you be willingly to do something? Yes ( ) No ( ) Don’t know ( )
2. If yes, what will you do?..............................................................................................
3. How safe to your health do you consider your current cooking fuel?

Highly unsafe( ) unsafe( ) Indifferent( ) safe( ) Highly safe( )

1. To what extent do you agree Government should ban the commercial usage of cooking fuel whose smoke have bad health effect?

Strongly Disagree ( ) Disagree ( ) indifferent ( ) Agree( ) Strongly Agree( )

1. If you are told that your current type of cooking smoke is bad for your health would you be willing to discard it and buy a less harmful smoke free stove?

Yes ( ) No ( ) Don’t know ( )

1. If No, Why:..............................................................................................................

..................................................................................................................................

1. If yes, how much money would you be willing to spend? .................................Naira
2. If you were told some cooking fuels are better for your health than the current one you are using, would you be willing to change? Yes ( ) No ( ) Don’t know ( )
3. If the best cooking fuel for your health was more expensive than the one you currently use, would you be willing to change? Yes ( ) No ( ) Don’t know ( )
4. If you are told that your current type of cooking smoke is bad for your health would you be willing to accept a less harmful smoke free stove if it was free of charge?

Yes ( ) No ( ) Don’t know ( )

1. Would you be willing to cook in a separate area? Yes ( ) No ( ) Don’t know ( )

**Section D: Beliefs**

1. Do you think some cooking fuels are better for your health than others?

Yes ( ) No ( ) Don’t know ( )

1. If yes, which one do you believe is the least harmful to health?

Biomass cooking fuel[ ] Kerosene [ ]Gas[ ] Electricity[ ] Others (specify)..................................

1. Why do you think so?..............................................................................................

...............................................................................................................................

1. If you were told some fuels are better for your health than others, would you be willing to change? Yes ( ) No ( )
2. If Yes, How much per week extra would you be ready to pay? ........................Naira
3. How much do you currently spend on buying cooking fuel in a week?.........................naira
4. Which type of cooking fuel do you prefer to use?

Biomass cooking fuel[ ] Kerosene [ ] Gas[ ] Electricity[ ]

Others specify.........................

1. Why? The food taste better Yes ( ) No ( )

No other alternative available Yes ( ) No ( )

Cheaper Yes ( ) No ( )

Don’t know of other alternatives Yes ( ) No ( )

It cooks faster Yes ( ) No ( )

It is safer than others Yes ( ) No ( )

Other reasons (specify) ..................................................................

1. Do you like to cook over an open fire with fire (except gas stove)?

Yes ( ) No ( ) (if No, skip to next Question)

1. Why do you like to cook over an open fire? (multiple answer)

The food taste better Yes ( ) No ( )

No other alternative available Yes ( ) No ( )

Cheaper Yes ( ) No ( )

Don’t know of other alternatives Yes ( ) No ( )

It cooks faster Yes ( ) No ( )

It is safer than others Yes ( ) No ( )

Other reasons (specify) ...........................................................................
